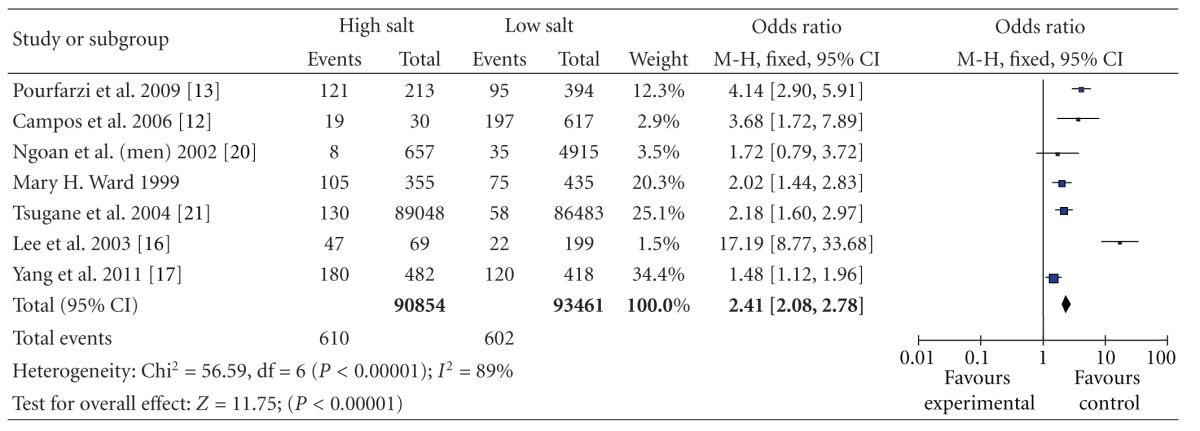# Association between Habitual Dietary Salt Intake and Risk of Gastric Cancer: A Systematic Review of Observational Studies

**DOI:** 10.1155/2012/808120

**Published:** 2012-10-22

**Authors:** Sheng Ge, Xiaohui Feng, Li Shen, Zhanying Wei, Qiankun Zhu, Juan Sun

**Affiliations:** Department of Clinical Nutrition, Shanghai Sixth People's Hospital, Shanghai Jiaotong University, Shanghai 200233, China

## Abstract

*Purpose.* Systematic reviews of case-control and prospective studies showed a positive association between habitual salt intake and gastric cancer. Given new studies published thereafter, we carried out a meta-analysis to assess the association between dietary salt intake and gastric cancer. *Methods.* Case-control studies and cohort studies published between January 1992 and January 2012 on PubMed and Embase were searched. We quantified associations between salt intake and gastric cancer with meta-analysis. *Results.* Eleven studies (7 case controls and 4 cohorts) finally were included in the meta-analysis (total population: *n* = 2076498; events: *n* = 12039). The combined odds ratio showed significantly positive association between high salt intake and gastric cancer compared with low salt intake (OR = 2.05, 95% CI [1.60, 2.62]; *P* < 0.00001). In subgroup meta-analysis, findings were slightly different when analyses were restricted to salty food intake (OR = 2.41, 95% CI [2.08, 2.78]; *P* < 0.00001) as well as in Asia (OR = 1.27 95% CI [1.22, 1.32]; *P* < 0.00001). There was no evidence that sample size, exposure assessment substantially influenced the estimate of effects. *Conclusions.* The systemic review supports the hypothesis that dietary salt intake is positively associated with the risk of gastric cancer.

## 1. Introduction

Dietary factors are important environmental risk determinants for cancer development. The role of dietary factors in gastric cancer was studied in the last 40 years and received particularly attention recently. It is difficult to establish a causal relationship between salt intake and gastric cancer due to methodological limitations among which is the valid measurement of accurate salt intake. Therefore, the conclusion is still unclear. In 2007, the Second Expert Report from the World Cancer Research Fund [1] supported the view that salt intake is significantly related to gastric cancer. From then on, two meta-analyses have been published addressing the association between salt intake and gastrointestinal metaplasia or gastric cancer. The study [2] on salt intake and gastrointestinal metaplasia revealed a positive trend but no statistical significance was observed. Another meta-analysis [3] included 7 prospective studies in total, and four out of seven were carried out in Japan. Although it demonstrated a positive association between salt consumption and incidence rate of gastric cancer, the limitation in geographic location prevents its generalization. Although prospective studies have more power in controlling confounders than case-control studies, the tendency of using baseline salt intake to represent the subsequent salt consumption causes inaccuracy in actual salt intake. Therefore, we carried out this systematic review to assess the relationship between habitual dietary salt intake and risk of gastric cancer.

## 2. Materials and Methods

### 2.1. Data Sources and Searches

This paper was planned, conducted, and reported according to the PRISMA statement [4]. We performed a systematic search for publications using MEDLINE and Embase databases (from 1992 to 2012). The following keywords were used in searching: “salt or sodium or salty or sodium chloride” and “gastric cancer or stomach cancer.” Moreover, we searched for the keywords in titles, abstracts and performed a manual search of references cited in the selected articles and published reviews.

### 2.2. Eligibility Criteria

Citations selected from the initial search were subsequently screened for eligibility. The list of references was independently screened by two reviewers. Cohort and case-control studies were included in the systematic review when all the following criteria were met: (1) original research addressing the association between the consumption of salt or salted foods and the occurrence of gastric cancer in humans; (2) prospective design; (3) adult population; (4) exposure defined as salt or salted foods by the authors of each study or including foods with high contents of salt as defined in the latest report of the World Cancer Research Fund and the American Institute for Cancer Research (processed foods such as processed meat; salty foods such as potato crisps or chips, salted nuts, and salty snack foods; salted foods such as bacon, sausages, and ham; salt-preserved foods such as salted meat, fish, vegetables, and fruits); (5) diagnosis of gastric cancer determined prospectively as outcome (gastric cancer incidence and/or mortality rate); (6) indication of the number of participants exposed and the rate or number of events in different categories of salt/sodium intake; (7) articles written in English.

### 2.3. Data Extraction and Quality Assessment

 We designed a data collection form before selecting eligible studies. The following data were extracted independently by two authors using a unified data form, the first author's full name, year of publication, country, gender, age, range of followup, study population, the events and mortality of gastric cancer, types of estimate of habitual sodium intake, and factors controlled and matched or adjusted variables in the design or data analysis. The results of the two authors were compared, and a consensus result would be achieved if there were any disagreement.

The Newcastle-Ottawa Scale [5] was used by two authors independently to evaluate the quality of the included studies. The Coding Manual for Case-Control Studies (an 8-stars system) was used to assess the quality of case-control studies, in which a study was judged on 3 broad perspectives as follows: (a) the selections of both study groups, (b) the comparability of study groups, and (c) exposure levels of study groups. The Coding Manual for Cohort Studies (a 9-star system) was used to assess the quality of cohort studies, as well as in case-control study. Each cohort study was judged on 3 broad perspectives as follows: (a) the selections of both study groups, (b) the comparability of study groups, and (c) outcome of the two study groups. The results of the two authors were compared, and a consensus result would be received if there was any disagreement.

### 2.4. Statistical Methods

We evaluated the differences between low salt consumption and high salt consumption on the occurrence of gastric cancer. The original data from the studies was used to calculate the summary OR with its 95% confidence intervals (CIs) in all analyses. A random-effects model was used to account for between-study heterogeneity and publication bias. 

The method of Mantel-Haenszel test [6] was used to assess the pooled OR and corresponding 95% confidence intervals (CIs) across studies. Forest plots were used to visually assess the results of Mantel-Haenszel test.

To assess the heterogeneity of ORs across studies, the Cochran Q statistic [7] (significance level of *P* < 0.10) and the *I* ^2^ statistic [8] (which quantifies the percentage of variation attributable to heterogeneity) were calculated. Subgroup analyses were carried out by region and food items to assess the relationship between salt intake and gastric cancer. The Begg test [9] and funnel plot [10] were used to assess the possibility of publication bias. 

## 3. Results

### 3.1. Literature Search

**Figure 1:**
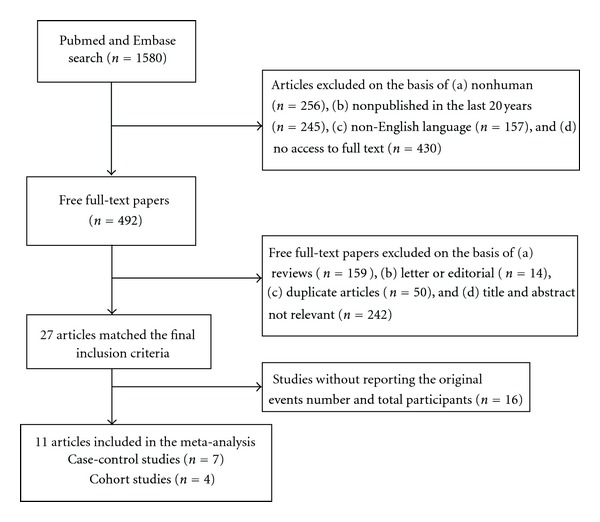
Process of study selection for salt intake and risk of gastric cancer.

Our search strategy yielded 1580 articles: 810 from Embase and 770 from Pubmed. A flow diagram that detailed the process is presented in Figure 1. The main details of the flow diagraph are as follows: after the first step, only 492 articles with full-text access are reserved. There were 159 reviews, 14 letters or editorial, and 50 duplicated articles among the 492 articles. 242 articles were excluded after we had reviewed titles and abstracts, and only 27 articles are reserved. 16 articles were excluded after reviewing the full text, because there were no original data. Finally, 11 studies (7 case-control studies [11–17] and 4 cohort studies [18–21]) were included in the meta-analysis.

### 3.2. Characteristics of the Included Studies

The characteristics of the studies included in our meta-analysis are showed in Table 1 [11–21]. The total number of participants was 2076498 [11–20, 20, 21]. The study design types were as follows: case-control study (*n* = 7 [11–17, 21]) and cohort study (*n* = 4 [18–21]). Studies were conducted in Japan (*n* = 3 [19–21]), Korea (*n* = 2 [16, 18]), China (*n* = 1 [17]), Spain (*n* = 1 [14]), Portugal (*n* = 1 [11]), Colombia (*n* = 1 [12]), Iran (*n* = 1 [13]), and Mexico (*n* = 1 [15]). Only 2 [18, 20] of the final 11 studies reported women and men independently. Total salt intake was used to assess sodium intake in 4 studies [11–13, 20]. Salted food was used in 6 studies [14–17, 20, 21]. Salt preference was used in the last 1 study [18].

The study quality scores were listed in Table 2: 6 studies got a score of 8 (4 case-control studies and 2 cohort studies), 5 studies got 7 points (3 case-control studies and 2 cohort studies).

### 3.3. The Risk of Gastric Cancer of High Salt Intake and Low Salt Intake

The multivariable-adjusted ORs for each study and combination of all studies for the high versus low categories of salt intake are shown in Table 3. The summary OR of all studies, using a random-effects model, showed that the high salt intake was significantly associated with a 105% greater risk of gastric cancer compared with low salt intake (OR = 2.05 95% CI [1.60, 2.62]; *Z* = 154.7; *P* < 0.00001). However, there was statistically significant heterogeneity across the studies (*P* < 0.01; *I* ^2^ = 92%), regarding the methods were used to evaluate the exposure, the food items evaluated, the consumption categories considered for analysis, and the degree of adjustment for possible confounders. Subgroup analysis was used for categorical variables.

A trend toward a direct association between salt intake and gastric cancer risk was detected in all 11 individual studies that were included in the meta-analysis and statistically significant in 9 of them.

### 3.4. Subgroup Analysis

Stratifying by geographic region, the pooled ORs of gastric cancer for the high versus low categories of salt intake were 1.15 (95% CI, [0.88, 1.52]) for studies conducted in Europe, there was no statistically significant heterogeneity among studies of salt intake in Europe (*P* = 0.19 and *I* ^2^ = 41%) (Table 4); and 1.27 (95% CI, [1.22, 1.32]) for studies conducted in Asia with stratification according to geographic region, and there was statistically significant heterogeneity among studies of salt intake in Asia (*P* < 0.00001 and *I* ^2^ = 95%) (Table 5). So separate analysis of the studies reporting further analyses was carried out to check for potential sources of heterogeneity that might explain the association between dietary salt intake and gastric cancer events in Japan. The OR was 2.61 (95% CI, [2.02, 3.38]) for studies conducted in Japan (Table 6).

We also conducted analyses that were stratified according to the food items, using studies that reported results on gastric cancer in relation to food items. The OR for salt was 1.20 (95% CI, [1.15, 1.26]), and there was statistically significant heterogeneity among these studies (*P* = 0.03 and *I* ^2^ = 67%) (Table 7). The OR for salty food was 2.41 (95% CI, [2.08, 2.78]), statistically significant heterogeneity was also found among these studies (*P* < 0.000 01 and *I* ^2^ = 89%) (Table 8). The results showed that different sources of salt intake (salt or food items) had different risks on gastric cancer. 

## 4. Discussion

Diet is considered to be associated with carcinogenesis. In this meta-analysis, we attempted to collect the evidence to identify the relationship between dietary salt intake and gastric cancer. Findings from the current study suggested that, compared with low salt intake, high salt intake showed significantly positive association with gastric cancer (overall OR = 2.05, 95% CI [1.60, 2.62]; *P* < 0.00001). However, there was a significant heterogeneity among the included studies. In subgroup analysis by category of salt intake, geographical regions, and sex, however, the significantly positive association was not changed.

Although we observed a positive association, there were many methodological limitations in human studies which prevent valid measurements used to assess salt consumption effectively [24]. For example, there are greater recall and selection biases in case-control studies because of their retrospective nature. In these studies, gastric cancer patients were more likely to change their dietary behavior as well as salty foods for their health. Then their earlier long-term dietary habit may have been strongly influenced by the recent diet. Because of different methods used to assess and report salt consumption across studies, we could not evaluate a dose-response relation between salt consumption and gastric cancer. We cannot be able to exclude the other confounding factors, such as mutagens in the salty foods or processed food.

The World Cancer Research Fund and the American Institute for Cancer Research published in 2007 a large systematic review and meta-analysis, which concerned the effect of salt in the development of gastric cancer [1]. A 17-article systematic review and meta-analyses on addressing the association between dietary salt exposure and gastric intestinal metaplasia received a positive association [2]. A meta-analysis (7 articles included) to assess the association between habitual salt intake and risk of gastric cancer in prospective studies also got a positive association [3]. However, there was a significant heterogeneity among the included studies in the three articles.

### 4.1. Potential Mechanisms

Several mechanisms which suggested that salt intake may increase gastric cancer risk have been postulated although there has been no consistent conclusion.

#### 4.1.1. The Destruction of the Mucosal Barrier

Intragastric high salt concentration destroys the mucosal barrier, through the increase of surface mucous cell mucin and decrease of gland mucous cell mucin [25], leading to inflammation and damage such as diffuse erosion and degeneration [21], produces atrophic gastritis and decreases the acidity of the stomach. It creates a condition favoring *H. pylori* infection [12].

#### 4.1.2. Intestinal Metaplasia

Intestinal metaplasia is also an important risk factor of gastric cancer. Mucosal damage in the stomach increases the rate of mitosis, leading to excessive cell replication [22] and hyperplasia of the gastric pit epithelium with increased potential for mutations [23]. Intestinal metaplasia characterized by the presence of caliciform cells in glands and in foveolar gastric mucosa was detected near regenerative hyperplasia foci high NaCl diets animals [26]. High salt intake will increase concentration of NaCl in the stomach; then it may accelerate the procedure of intestinal metaplasia and increase the risk of gastric cancer in the future.

#### 4.1.3. Hypergastrinemia

Gastrin itself may mediate epithelial cell growth in *H. pylori*-colonized mucosa [27] and induce hypergastrinemia [28]. Chronic hypergastrinemia can synergize with *Helicobacter* infection and lead to eventual parietal cell loss and progression to gastric cancer [29].

#### 4.1.4. *H. pylori*

*H. pylori* is one of the important recognized risk factors of gastric cancer. The damage caused by salt may also increase gastric *H. pylori *colonization. *H. pylori* responds to changes in the concentration of NaCl in its environment in such a way that growth, cell morphology, survival, and virulence factor expression are all altered by increased salt concentration [30]. Elevated salt concentrations result in alterations in expression of the virulence factor CagA in *H. pylori* strain 26695 and enhance the ability of CagA to translocate into gastric epithelial cells and enhance the ability of *H. pylori* to alter gastric epithelial cell function [31]. 

#### 4.1.5. Endogenous Mutations

Salt may also directly damage gastric mucus, improve inflammatory responses of the gastric epithelium [32], which may increase epithelial cell proliferation as part of the repair process, potentiate the action of carcinogens [33], and increase the probability of endogenous mutations [34]. 

#### 4.1.6. Exposure to Carcinogens

High dietary salt intake damages the stomach mucosa that protects the stomach and increases the susceptibility of the mucosal cells to carcinogens from foods, such as N-nitroso compounds. And its repair is associated with inflammatory changes [35] and leads to cell death [36]. But the studies included in the current meta-analysis did not report the potential carcinogens of the salty foods or processed food. Future observational studies should pay more attention in this area. 

### 4.2. Limitations

Despite these advantages, the current meta-analysis, however, had limitations. 

First, the majority of included studies used questionnaire to assess habitual salt intake which had limited value [37]. Only few studies used 24 h urinary sodium excretion as indictor of salt consumption which is recommended by the World Cancer Research Fund as the best measurement of salt intake[38].The information derived from the questionnaire is subjective, qualitative and had not covered all the sources of sodium intake [39]. In most studies, the consumption of salted food which is high in salt and nitrites as well was recorded as a source of sodium intake. It is well known that nitrite is a mutagen that is closely related to gastric carcinogenesis [40, 41]. These methodological limitations compromised the association between salt intake and gastric cancer, either toward exaggeration or underestimation of risk estimates.

Second, due to the huge heterogeneity of the related data presented in the studies, the number of studies involved in the meta-analysis was relatively small. Therefore, subgroup analyses were difficult to perform.

Third, the current meta-analysis is unable to rule out the possible influence of confounding factors on the revealed association. Some confounders were inherent in the included studies. Although each study recruited some known risk factors for adjustment for gastric cancer, these covariates were not consistent and unknown confounders such as mutagens in salted foods cannot be excluded as a potential explanation for the observed findings.

Fourth, the cutoff values corresponding to the low and high categories for salt intake variedwidely among the studies, which might also affect the obtained results.

In conclusion, the overall current literature on dietary salt intake and the risk of gastric cancer suggested significantly positive association. Due to the nature of the association, more well-designed prospective studies that use unified measures of dietary salt intake are needed to fully characterize such an association, and it is impossible to perform a large randomized, controlled clinical trial to clarify the cause-effect relationship. Therefore, future observational study with recommended salt assessment method and maximized exclusion of confounders from salted foods is necessary.

## Figures and Tables

**Table 1:** The characteristics of the studies included in the meta-analysis.

First author							Gastric cancer (*n*)		Type of estimate of habitual sodium intake	Factors controlled for in multivariate analysis
	Publication year	Location	Study period	Sex	Age (years)	Study population (*n*)	Events	Mortality		
Peleteiro [11]	2011	Portugal	2001–2006	F/M	18–92	1071	422	~	Total salt intake	Age, gender, education, smoking, *H. pylori* infection, and total energy intake

Campos [12]	2006	Colombia	2000–2002	F/M	49–75	647	216	30	Total salt intake	Age, gender, and hospital

Pourfarzi [13]	2009	Iran	2003–2005	F/M	65.46 ± (11.5)	611	217	~	Total salt intake	Age, gender, residence, education, and family history on cancer

Kim [18]	2010	Korea	1996–2003	F/M	30–80	2248129	12393	~	Salt preference	Age, sex, BMI, smoking habits, alcohol consumption, physical activity, and family history of cancer

Ramón et al. [14]	2002	Spain	1986–1989	F/M	32–80	305	165	12	Smoked and pickled foods, salt	Age, gender

Shikata [19]	2006	Japan	1998–2002	F/M	>40	2467	93		Total salt intake	Age, sex, *Helicobacter pylori* infection, atrophic gastritis, medical history of peptic ulcer, family history of cancer, body mass index, diabetes mellitus, total vitamin B2, vitamin C and dietary fiber intake, smoking habits and dietary factors intake of total energy, total protein, carbohydrate, vitamin B1, cholesterol, physical activity, and alcohol

Ngoan [20]	2002	Japan	1986–2001	F/M	>15	13250	116	116	Pickled food	Age, sex, smoking, and other dietary factors

Ward [15]	1999	Mexico	1989–1990	F/M	~	972	220	~	Salty snacks	Age, gender, and total calories

Tsugane [21]	2004	Japan	1990–2001	F/M	40–59	39065	486	361	Salted food consumption (miso soup, pickled vegetables, salted fish roe, salted fish preserves, and dried or salted fish)	Age, gender, cigarette smoking, fruit and vegetable intake, drinking history, and personal medical history

Lee [16]	2003	Korea	1999.3–1999.9	F/M	~	268	69	~	Salt-fermented fish	Age, sex, education, family history of gastric cancer, smoking, alcohol drinking, and *H. pylori* infection

Yang [17]	2011	China	2006–2010	F/M	40–75	900	300		Salted food (meat and fishes, pickled vegetable)	Age, sex, smoking, drinking, fresh fruit, and fresh vegetables

**Table 2:** The study quality scores of the studies included in meta-analysis.

First author, year of publication (reference)	Objects selection				Comparability	Exposure/result			Total quality scores
	Adequate definition/exposed cohort representat-iveness	Representativeness of cases/nonexposed cohort	Controls selection/exposure ascertainment	Controls definition/outcome not present		Exposure ascertainment/outcome assessment	cases and controls ascertainment method/followup length	Nonresponse rate/adequate followup	
Campos, 2006 [12]	*⋆*	*⋆*	0	*⋆*	*⋆* *⋆*	*⋆*	*⋆*	0	7
Tsugane, 2004 [21]	*⋆*	*⋆*	*⋆*	*⋆*	*⋆* *⋆*	*⋆*	*⋆*	0	8
Peleteiro, 2011 [11]	*⋆*	*⋆*	*⋆*	*⋆*	*⋆* *⋆*	*⋆*	*⋆*	0	8
Pourfarzi, 2009 [13]	*⋆*	*⋆*	*⋆*	*⋆*	*⋆* *⋆*	*⋆*	*⋆*	0	8
Yang, 2011 [17]	*⋆*	*⋆*	*⋆*	*⋆*	*⋆* *⋆*	*⋆*	*⋆*	0	8
Shikata, 2006 [19]	0	*⋆*	*⋆*	*⋆*	*⋆* *⋆*	*⋆*	*⋆*	0	7
Ward, 1999 [15]	0	*⋆*	*⋆*	*⋆*	*⋆* *⋆*	*⋆*	*⋆*	0	7
Ngoan, 2002 [20]	0	*⋆*	*⋆*	*⋆*	*⋆* *⋆*	*⋆*	*⋆*	0	7
Ramón et al., 1993 [14]	*⋆*	*⋆*	*⋆*	*⋆*	*⋆* *⋆*	*⋆*	*⋆*	0	8
Lee, 2003 [16]	*⋆*	*⋆*	*⋆*	*⋆*	*⋆* *⋆*	*⋆*	*⋆*	0	8
Kim, 2010 [18]	*⋆*	*⋆*	*⋆*	*⋆*	*⋆* *⋆*	*⋆*	*⋆*	0	8

**Table 3:** High versus low categories of salt and gastric cancer.

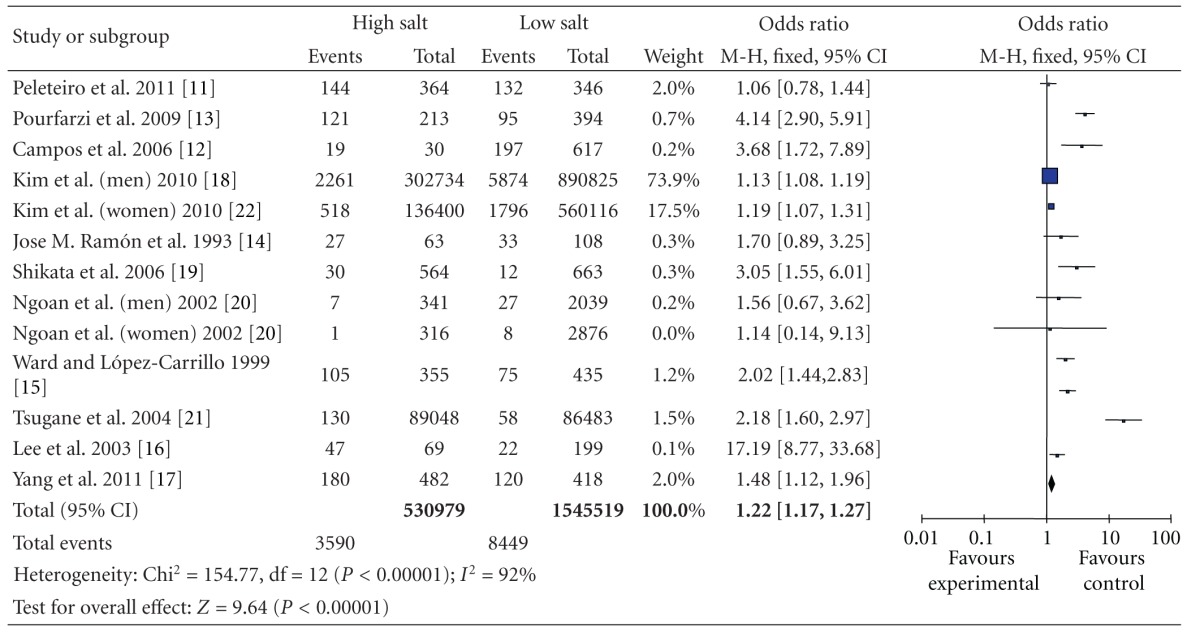

**Table 4:** High versus low categories of salt and gastric cancer in Europe.

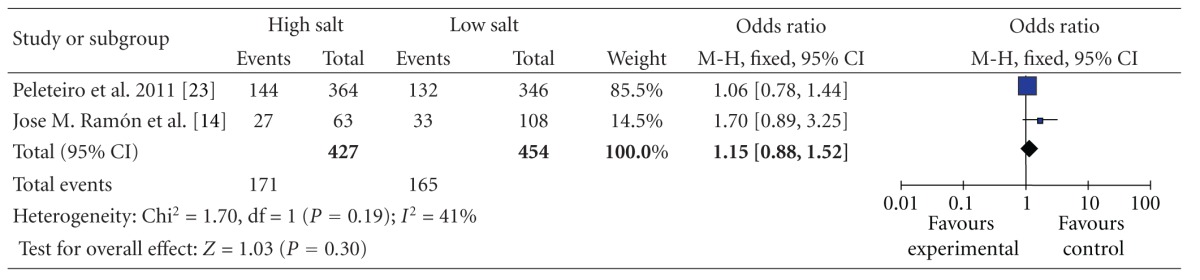

**Table 5:** High versus low categories of salt and gastric cancer in Asia.

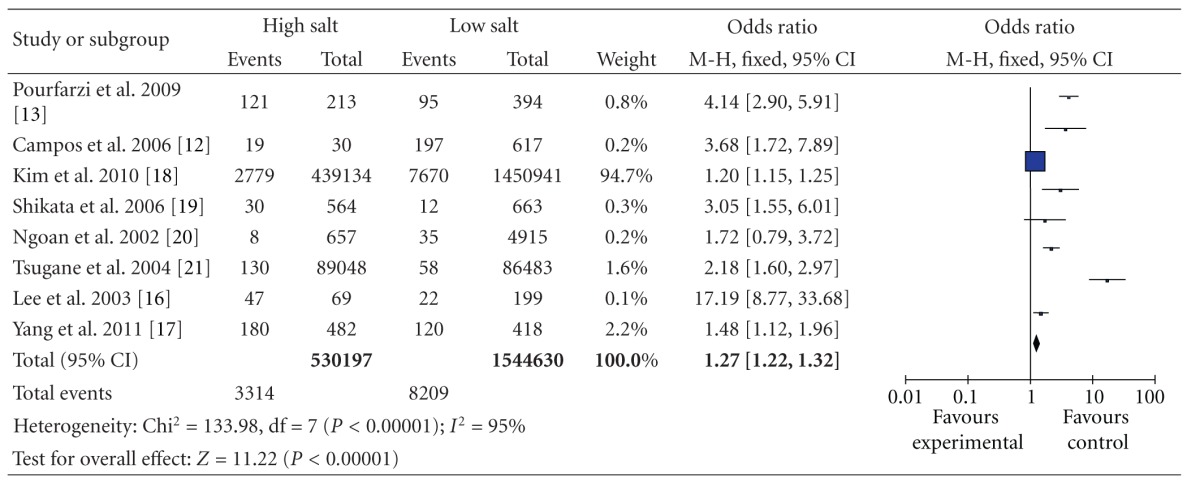

**Table 6:** High versus low categories of salt and gastric cancer in Japan.

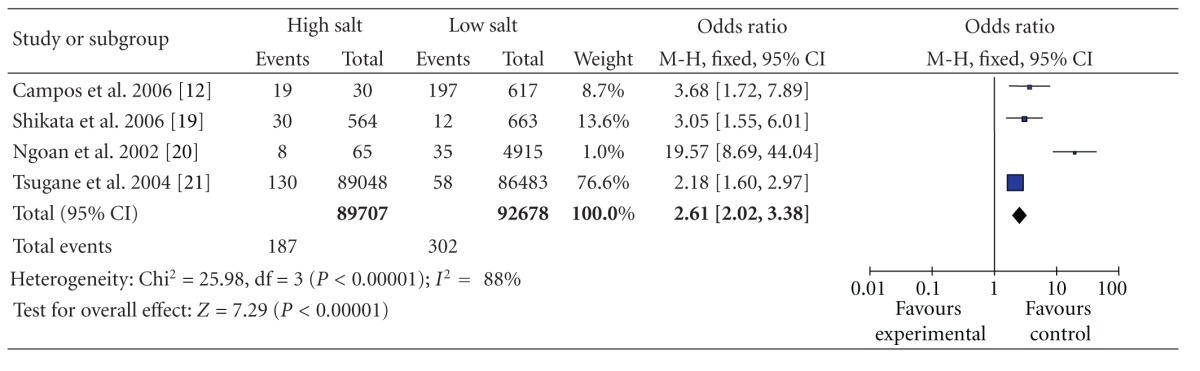

**Table 7:** High versus low categories of salt and gastric cancer through salt.

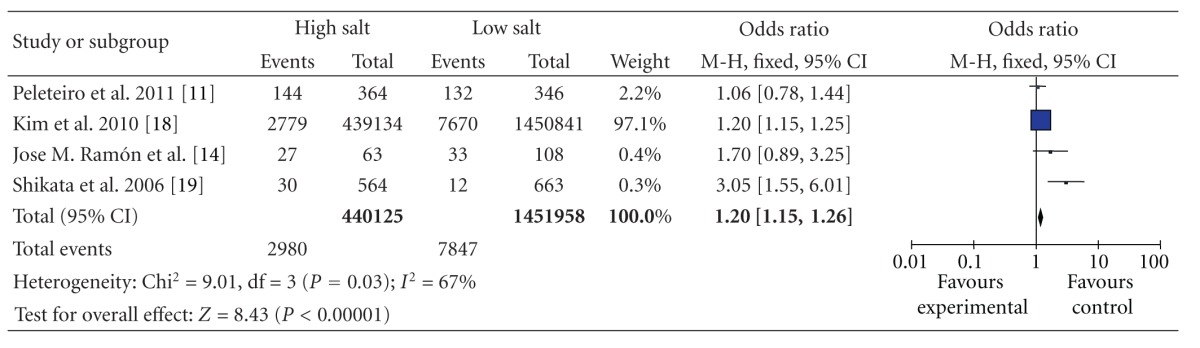

**Table 8:** High versus low categories of salt and gastric cancer through salty food.